# Supplementary material for: Predicting visual function by interpreting a neuronal wiring diagram
Source: Nature. 2024 Oct 2;634(8032):113–23. doi: 10.1038/s41586-024-07953-5 (PMC11446822; doi:10.1038/s41586-024-07953-5)
Supplement: Supplementary file 6 — CRF and ERF predictions for individual TmY4 and TmY9 cells. Analogous to Supplementary Data 3, but for TmY target types. Shown are the top four monosynaptic pathways, the strongest pathway passing through each of the top ten intermediary types (ranking from Extended Data Fig. 7), and the trisynaptic pathway Tm1–TmY–Dm3–TmY (see the section entitled Prediction of spatial normalization). [file 41586_2024_7953_MOESM6_ESM.zip › DataS4/TmY9qΓèÑ/720575940628411722.pdf]

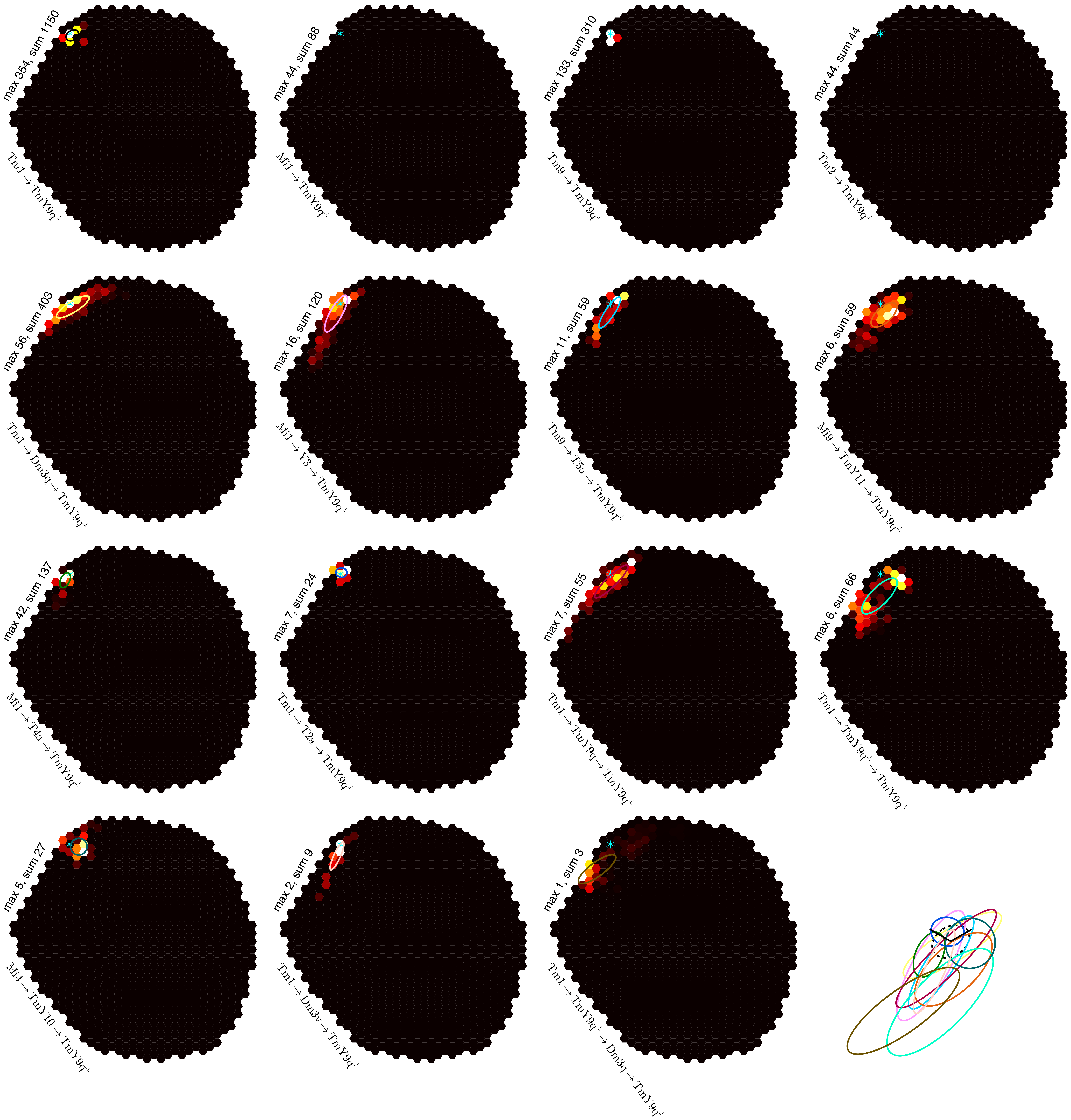
max 354, sum 1150
Tm1 → TmY9q⊥
max 44, sum 88
Mi1 → TmY9q⊥
max 133, sum 310
Tm9 → TmY9q⊥
max 44, sum 44
Tm2 → TmY9q⊥
max 56, sum 403
Tm1 → Dm3q → TmY9q⊥
max 16, sum 120
Mi1 → Y3 → TmY9q⊥
max 11, sum 59
Tm9 → T5a → TmY9q⊥
max 6, sum 59
Mi9 → TmY11 → TmY9q⊥
max 42, sum 137
Mi1 → T4a → TmY9q⊥
max 7, sum 24
Tm1 → T2a → TmY9q⊥
max 7, sum 55
Tm1 → TmY9q → TmY9q⊥
max 6, sum 66
Tm1 → TmY9q⊥ → TmY9q⊥
max 5, sum 27
Mi4 → TmY10 → TmY9q⊥
max 2, sum 9
Tm1 → Dm3v → TmY9q⊥
max 1, sum 3
Tm1 → TmY9q⊥ → Dm3q → TmY9q⊥
